# ﻿A new synonym of *Rhododendronleishanicum* (subg. *Hymenanthes*) from China (Ericaceae)

**DOI:** 10.3897/phytokeys.243.121263

**Published:** 2024-06-14

**Authors:** Sheng Chen, Lei Hao, Yi-Hui Deng, Cheng-Hua Yang, Jun-Mei Yuan, Jia-Xin Lou, Yun-Song He, Xiang Chen

**Affiliations:** 1 Institute of Biology, Guizhou Academy of Sciences, Guiyang 550009, Guizhou, China Institute of Biology, Guizhou Academy of Sciences Guiyang China; 2 Liuzhou Forestry Research Institute, Rongshui 545300, Guangxi, China Liuzhou Forestry Research Institute Rongshui China; 3 Guizhou Academy of Forestry, Guiyang 550005, Guizhou, China Guizhou Academy of Forestry Guiyang China

**Keywords:** Morphology, new synonym, *Rhododendron*, *Rhododendronleishanicum*

## Abstract

Based on a critical examination of type specimens, images of living plants, and the literature has shown *Rhododendronoligocarpum* to be conspecific with *R.leishanicum*. Although slight variations in corolla colour exist amongst different populations of *R.oligocarpum*, it does not serve as a key distinguishing trait. Therefore, we reduced *R.oligocarpum* to a synonym of *R.leishanicum*, and recommend placing it in Subsection Maculifera.

## ﻿

*Rhododendron* L. which contains ca. 1200 species, is the largest genus of Ericaceae (Chamberlain et al. 1996; MacKay and Gardiner 2017). This genus is widely distributed in Asia, Europe and North America, of which the great majority (ca. 900) occurs in China and the Malaysian archipelago, the centres of diversity being in the Himalayas and South East Asia (Fang et al. 2005; Brown et al. 2006; Gibbs et al. 2011; Liu et al. 2020). When we examined the type specimens of *Rhododendron* from Guizhou Province, China, *R.leishanicum* W. P. Fang et S. S. Chang ex D. F. Chamb. and *R.oligocarpum* W. P. Fang et S. S. Chang were found to be morphologically very similar and identified as very confusing in terms of leaf, flower, and fruit characters.

**Figure 1.**
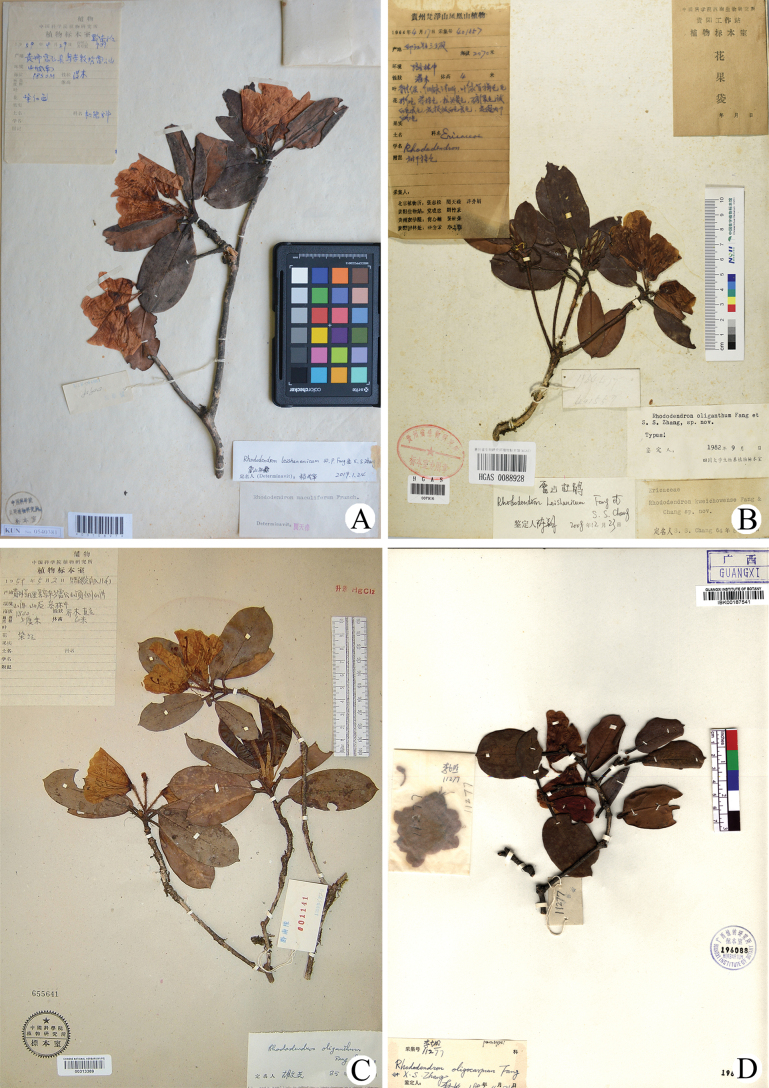
*Rhododendronleishanicum* and *R.oligocarpum***A** isotype of *R.leishanicum***B** holotype of *R.oligocarpum***C, D** paratype from Leigong Mountain and Maoer Mountain of *R.oligocarpum*, respectively.

*Rhododendronleishanicum* was originally described by Chamberlain (1982), based on a single collection, Austro-Guizhou Exped 909, from Leigong mountain in Lei Shan Xian, Guizhou Province, China (Fig. 1A). In the protologue, Chamberlain placed *R.leishanicum* in the Subsection Williamsiana. The next year, *R.oligocarpum* was described by Fang (1983), based on six collections (including Z. S. Zhang et al. 401557 (Types: HGAS0088928, IBSC0481928, PE01297915, PE01297916), Z. S. Zhang 58 (HGAS007915), T. H. Tu 31739 (SZ0036179), from Fanjing Mountain; Austro-Guizhou Exped 1411 (PE00312607, PE00313389, KUN540382), from Leigong Mountain and G. Z. Li 6211 & 11277 (IBK00187538, IBK00187539 & IBK00187541, IBK00187559), from Maoer Mountain) from three different origins in China (Fig. 1B–D). According to the protologue, Fang placed *R.oligocarpum* in the Subsection Maculifera. Meanwhile, *R.leishanicum* was again described as a new species by Fang in the same protologue. It is worth noting that a paratype of *R.oligocarpum*, Austro-Guizhou Exped 1141, was collected from Leigong Mountain (Fig. 1C). However, the relationship between *R.oligocarpum* and *R.leishanicum* was not mentioned by Fang when he described *R.leishanicum* as a new species again in the publication in 1983.

**Figure 2.**
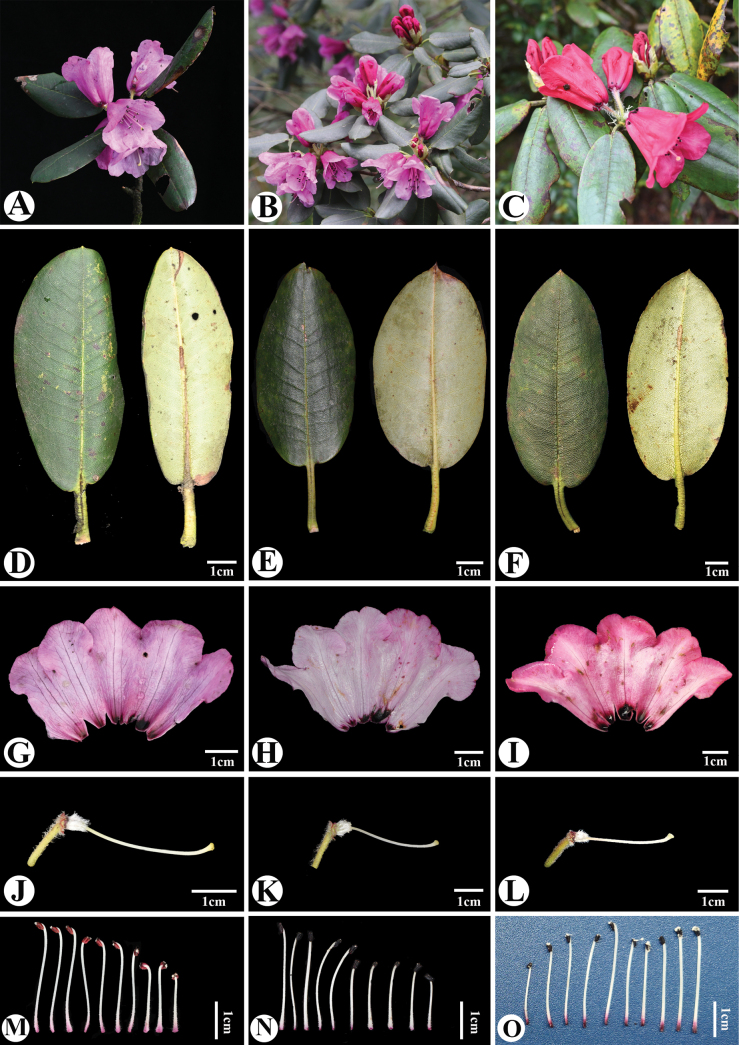
Comparison of *Rhododendronleishanicum* and *R.oligocarpum***A, D, G, J, M***R.leishanicum* from Leigong mountain **B, E, H, K, N***R.oligocarpum* from Fanjing mountain **C, F, I, L, O***R.oligocarpum* from Maoer mountain **A–C** branchlets with flowers **D–F** adaxial and abaxial surface of leaf **G–I** corolla with deep purple basal spots **J–L** pedicel, Calyx, ovary and style indumentum **M–O** stamens side view.

According to critical examination of the type specimens and morphological comparisons of *R.leishanicum* and *R.oligocarpum*, we found that both species have setose young branches and petioles, with small apiculate leathery leaves, racemose-umbellate inflorescences, corolla campanulate, with deep purple basal spots, pedicel, ovary and filament with identical indumentum (Fig. 2). It is worth noting that during our field surveys of populations of these two species, we observed a certain difference in colour of the corolla between the Guangxi population of *R.oligocarpum* and those in Guizhou. However, variation in corolla colour is known to occur within species of the genus *Rhododendron* (Jin 2006; Zhou 2008); hence, the differences in corolla colour between them fall within a normal range of variation. Therefore, we place *R.oligocarpum* in synonymy with *R.leishanicum* according to ICN rules (Turland et al. 2018).

Based on the setose branchlets and petioles, the small apiculate leaves, the campanulate corolla and the glabrous style, we suggest placing *R.leishanicum* in the Subsection Maculifera.

## ﻿Taxonomy treatment

### ﻿ Rhododendron leishanicum

Taxon classificationPlantaeEricalesEricaceae

W. P. Fang & S. S. Chang ex D. F. Chamb.

EF5E0E69-F058-5A04-8F21-C1477F5F62CB

#### Type.

China. Guizhou province: Leishan Xian, Leishan, 1850 m elev, 29 April 1959, Austro-Guizhou Exped 909 (holotype: SZ; isotype: HGAS007912!; KUN0540381!).

### ﻿ Rhododendron oligocarpum

Taxon classificationPlantaeEricalesEricaceae

W. P. Fang & S. S. Chang syn. nov.

941FBE28-93BD-5C73-BEC0-7F256EB42582

#### Type.

China. Guizhou province: Yinjiang county, Sanduodian, 2070 m elev, 14 april 1964, Z. S. Zhang et al. 401557 (holotype: HGAS0088928!; isotypes: IBSC0481928!; PE01297915!, PE01297916!; paratype: HGAS007915!; IBK00187538!, IBK00187539!, IBK00187541!, IBK00187559!; PE00312607!, PE00313389!; KUN540382!; SZ0036179!).

#### Description.

Shrubs or small trees, 3–6 m tall; bark grey, dehiscent on drying; **branchlets** terete, setose when young, gradually glabrescent. **Petiole** rounded abaxially, furrowed adaxially, 5–15 mm, densely setose; **leaf** blade leathery, elliptic or obovate, 4–8 × 2–4 cm; base rounded or subcordate; margin revolute, apex rounded, with small apiculate; adaxial surface green, abaxial surface pistachio; mid-rib impressed abaxially, grooved adaxially, glabrous or sometimes sparsely hairy; lateral veins 11–15-paired, slightly conspicuous on both surfaces. **Inflorescence** racemose-umbellate, 3–5-flowered; rachis 5–7 mm, tomentose. **Pedicel** 1–2 cm, densely setose-tomentose; **Calyx** small, discoid, lobes 5, triangular, tomentose, 1–2 mm. **Corolla** campanulate, purple-red to rose-red, with deep purple basal spots, 3–3.5 cm; lobes 5, suborbicular, ca. 1.5 × 2 cm, apex emarginate; **stamens** 10, unequal, 1–3 cm, filaments puberulent at base; **ovary** cone-shaped, 4–5 mm, densely densely setose and tomentose; **style** 2.5–3 cm, glabrous, stigma slightly expanded. **Capsule** cylindrical, 20–25 × 7 mm, rough. Flowering from April to May and fruiting from September to October.

#### Distribution and habitat.

*Rhododendronleishanicum* is distributed in Eastern Guizhou and North-eastern Guangxi. It grows in Thickets at 1800–2500 m a.s.l.

#### Specimens examined.

**China. Guizhou**: Qiandongnan Prefecture, Leigong Mountain, *P. L. Song 1016* (GYBG barcode 0002282, 0002283), *4407* (CCNU barcode 9018123), *4717* (CCNU barcode 9018122); Tongren City, Fanjing Mountain, Z. S. Zhang et al. 401673 (IBSC barcode 0481934), *401317* (HGAS barcode 0088940, IBSC barcode 0481929), *400681* (HGAS barcode 0088933), *400610* (HGAS barcode 0088939), *Z. P. Jian 32039* (HGAS barcode 0088938), *Wuling Mountain Expedition 731* (GFS barcode 0007355, KUN barcode 0339467, 0339468, PE barcode 00258535), *1348* (GFS barcode 0007356, KUN barcode 0339469). **Guangxi**: Guilin City, Maoer Mountain, *G. Z. Li 11272* (IBK barcode 00187540), *12378* (IBK barcode 00187542), *12084* (IBK barcode 00187561), *F. X. Jin 1066* (HTC barcode 0010523, 0010524, 0010525), *J. X. Zhong 83311* (IBK barcode 00187551, 00187552, IBSC barcode 0481913), *83523* (IBK barcode 00187560), *81647* (IBK barcode 00187566, 00187567), *L. M. Gao 20077* (KUN barcode 0767388, 0767389).

## Supplementary Material

XML Treatment for Rhododendron leishanicum

XML Treatment for Rhododendron oligocarpum

## ﻿Additional information

### Author ORCIDs

Sheng Chen https://orcid.org/0000-0002-2727-6504

Lei Hao https://orcid.org/0009-0001-0276-6523

Yi-Hui Deng https://orcid.org/0009-0006-2821-0247

Chen-Hua Yang https://orcid.org/0000-0002-5941-676X

Jun-Mei Yuan https://orcid.org/0009-0007-1861-5790

Jia-Xin Lou https://orcid.org/0009-0001-0276-6523

Yun-Song He https://orcid.org/0009-0002-8791-558X

Xiang Chen https://orcid.org/0009-0008-0773-4978

### Data availability

All of the data that support the findings of this study are available in the main text.
